# Disparities in the uptake of learning disability annual health checks: a secondary analysis of primary care data

**DOI:** 10.3399/BJGP.2026.0116

**Published:** 2026-08-11

**Authors:** Daniel Sutherland, Benjamin Costello, Umesh Chauhan, Samantha Flynn, Kylie M Gray, Joanne Parsons, Rachel Spencer, Hayley Crawford

**Affiliations:** 1 Warwick Medical School, University of Warwick, Coventry, UK; 2 University of Lancashire, Preston, UK; 3 Centre for Research in Intellectual and Developmental Disabilities, University of Warwick, Coventry, UK; 4 Intellectual Disabilities Research Institute (IDRIS), School of Social Policy and Society, University of Birmingham, Birmingham, UK; 5 Department of Psychiatry, School of Clinical Sciences at Monash Health, Monash University, Australia, Clayton, Australia; 6 Health Services Management Centre, School of Social Policy, University of Birmingham, Birmingham, UK

**Keywords:** annual health check, general practice, intellectual disability, learning disability, primary health care

## Abstract

**Background:**

People with intellectual disabilities (referred to as learning disabilities in the UK) face considerable health inequalities. One attempt to address these in England has been through incentivising general practices to complete annual health checks with people with a learning disability aged ≥14 years.

**Aim:**

To examine recent trends in learning disability registers and the uptake of learning disability annual health checks in England.

**Design and setting:**

Secondary analysis of publicly available general practice data.

**Method:**

NHS England data relating to the learning disabilities annual health check scheme from January 2022 to November 2025 were compiled and analysed.

**Results:**

The number of 14–17-year-olds and adults on learning disability registers increased during this period by 30.49% and 14.53%, respectively. The most recent estimates indicate that around 0.588% (*n* = 374 763/63 766 671) of patients are on learning disability registers, with approximately 0.526% (*n* = 355 102/63 654 156) aged ≥14 years and eligible for annual health checks. The uptake of annual health checks increased slightly from 68.08% (*n* = 15 940/23 414) to 71.58% (*n* = 19 217/26 846) among 14–17-year-olds, and from 79.07% (*n* = 226 701/286 714) to 80.71% (*n* = 248 449/307 827) among adults. Uptake was lowest in the South West and East of England, and highest in London, but was similar across deciles of patient deprivation and practice size.

**Conclusion:**

Ascertainment of people with a learning disability by primary care services appears to be improving but remains between one-third and one-quarter of prevalence estimates. The uptake of annual health checks among 14–17-year-olds continues to lag behind that of adults.

## How this fits in

GP learning disability registers are growing year-on-year. Annual health check uptake is only fractionally improving despite incentive schemes and many checks are only completed in the lead up to Quality and Outcomes Framework reporting each year. Uptake of health checks among 14–17-year-olds continues to lag behind adults and there are substantial gaps between geographic regions of England. Uptake does not appear to be associated with socioeconomic deprivation or practice size.

## Introduction

Intellectual disabilities (referred to as learning disabilities in the UK and hereafter) involve impairments in cognitive ability and adaptive behaviour originating during the developmental period.^1^ People with a learning disability experience considerable health inequalities including earlier deaths and higher levels of multimorbidity compared with the general population.^2,3^ Furthermore, people with a learning disability often receive poorer quality health care. Their deaths are almost twice as likely to be considered avoidable,^2^ health problems may be missed because of diagnostic overshadowing,^4^ and healthcare professionals describe a range of challenges in working with people with a learning disability; such as lack of specialist knowledge and training.^5–7^ To address these issues, the NHS in England introduced a learning disability annual health check (AHC) scheme in 2008. In England, patients are registered with a single general practice through which they receive primary health care. The AHC scheme is a Directed Enhanced Service, meaning general practices are incentivised to provide AHCs to patients who are aged ≥14 years and recorded on their practice’s learning disability register: a list of patients known to have a learning disability that is used to facilitate appropriate care. The AHC is also supposed to involve the development of a health action plan that outlines steps to improve an individual’s health. The AHC scheme’s cycle runs from April of each year to the following March in alignment with the Quality and Outcomes Framework (QOF) — a general practice pay-for-performance scheme. Eligible patients are identified through learning disability registers but only around 0.5% of all patients are included on these registers — substantially fewer than the estimated 1.5%–2% prevalence of people with learning disabilities in England.^8,9^

AHCs have been shown to improve health care for people with a learning disability. In particular, research with adults has found that learning disability AHCs are associated with increased health assessments, diagnoses of previously unidentified problems, initiation of treatment, and a reduction in hospital admissions for ambulatory care sensitive conditions.^10–12^ Despite these benefits, uptake remains suboptimal. Many patients do not receive an AHC each year and, given the low coverage of learning disability registers, only a minority of those who are potentially eligible for the scheme actually receive AHCs. Uptake of AHCs among 14–17-year-olds has also lagged behind adults, with only 58.4% of adolescents on learning disability registers accessing an AHC in 2021–2022.^13^

There are many factors that could influence practice-level differences in AHC uptake. These include neighbourhood socioeconomic deprivation, which is consistently associated with poorer health outcomes in both the general population^14^ and in people with a learning disability.^2^ Geographical factors, such as regional service landscapes, may also contribute to primary care access and practice-level differences in AHC uptake.^15^ In addition, general practices vary dramatically in size and these differences may alter their approach to maximising uptake of Directed Enhanced Services such as AHC uptake.^16,17^ Understanding how uptake varies across practice-level factors such as these could help to better evaluate the implementation of the scheme and inform strategies to reduce inequalities.

Given these concerns regarding the inadequate coverage of learning disability registers, suboptimal AHC uptake, and uncertainty regarding factors influencing uptake, the aim of this study was to examine national English primary care data to identify trends in learning disability registers and AHC uptake among adolescents and adults. This study also considered the implications of these data for policy, practice, and research.

## Method

### Study design

This was a longitudinal descriptive study analysing public data from the learning disabilities health check scheme dataset^18^ and the English public health platform Fingertips.^19^

### Data sources

Monthly data on learning disability health checks were obtained from NHS England’s learning disability health check scheme dataset. This includes all practices that participate in the scheme (>95% of all practices) and is automatically extracted from practices’ IT systems. The dataset contains practice-level counts for indicators relating to the learning disability register and AHCs (see Supplementary Table S1 for full indicator definitions).

Data extraction began from April 2022 to coincide with the 2022–2023 QOF year and a new data structure and set of indicators. To account for a discontinuity in the number of patients on learning disability registers observed in April 2022, the authors of the current study manually added data from January to March 2022. The final dataset included data up to November 2025 and included 6452 general practices.

Practice patient deprivation was measured using patient-weighted Index of Multiple Deprivation (IMD) deciles derived from the 2025 IMD dataset. The IMD ranks small English areas called lower-layer super output areas (LSOAs) based on indicators for seven domains of local deprivation.^20^ NHS England data were extracted from the April of each year (the start of the QOF year) on the number of patients in each LSOA at each general practice. These were used to compute a mean patient-level IMD score, which was then categorised into deciles for reporting and analysis. This was successfully computed for 99.98% (*n* = 18 791/18 795) of practice years.

Practice size was based on the total number of registered patients and was extracted from Fingertips (a public health data platform provided by the Department of Health and Social Care) and linked using general practice codes. Practice size data were linked for 98.81% (*n* = 6375/6452) of practices. Similarly, data were extracted from Fingertips on the percentage of total QOF points that practices received each year. This was successfully linked for 99.72% (*n* = 6434/6452) of practice years.

The NHS digital learning disabilities health check scheme dataset only includes data for the numbers of patients aged ≥14 years who are on learning disability registers. The study therefore also reports estimates from Fingertips for the number of patients of any age on learning disability registers.^21^

### Data processing and analysis

The data were processed and analysed in R (version 4.4.3). The complete code can be found at https://osf.io/cf9kw. Descriptive analyses were conducted of each indicator including the number of patients on learning disability registers, and monthly and annual AHC uptake. As the study analysed data from nearly all general practices in England, inferential analyses based on population sampling were deemed inappropriate. However, Wilson 95% confidence intervals are included, when appropriate, to reflect the precision of estimates and to characterise imprecision arising from random variation in underlying processes. To accurately characterise uptake, percentage uptake figures include patients recorded as having declined a health check in the denominator, in contrast to NHS Digital performance metrics that exclude these patients.

## Results

### Number of patients on learning disability registers

First, changes in the number of patients on learning disability registers were examined from January 2022 to November 2025. As shown in Figure 1, the number of 14–17-year-olds on registers increased by 30.49% from 21 190 people in January 2022 to 27 650 in November 2025, and the number of adults increased by 14.53% between January 2022 and November 2025 from 276 023 to 316 116. By comparison, Fingertips data suggest that between 2022 and 2025, the number of registered patients in England aged <18 years and aged ≥18 years rose by 7.19% and 2.55%, respectively, highlighting that these increases cannot be accounted for by increased numbers of patients alone.

**Figure 1.**
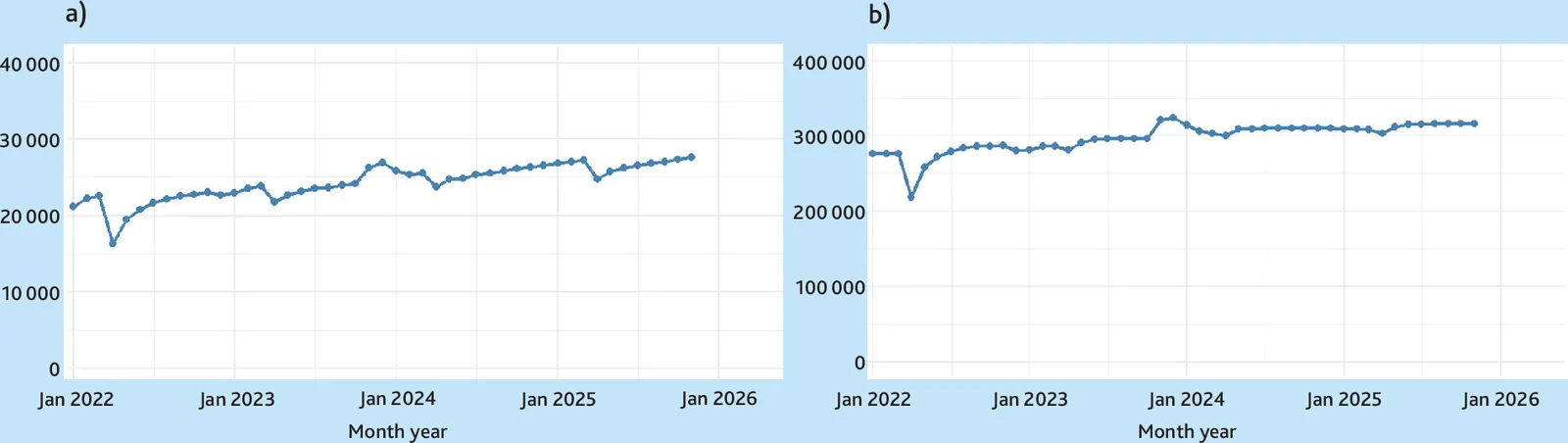
The number of a) 14–17-year-olds and b) adults on learning disability registers over time. Jan = January.

### Percentage of patients on learning disability registers

The percentage of patients on learning disability registers was calculated. At the end of the 2022–2023 QOF year, 0.500% of total registered patients (*n* = 306 877/61 435 922) were aged ≥14 years and on learning disability registers, and therefore eligible for learning disability AHCs. This increased to 0.521% of patients (*n* = 328 772/63 109 035) in the 2023–2024 QOF year, and 0.526% (*n* = 335 102/63 654 156) in 2024–2025. The learning disability AHC dataset does not include data for patients aged <14 years. However, total numbers of patients on learning disability registers (of all ages) are reported in Fingertips.^21^ These data indicate that in 2022–2023, 0.558% of patients (*n* = 347 840/62 378 057) were on learning disability registers, and this increased to 0.580% (*n* = 366 842/63 213 403) in 2023–2024, and 0.588% (*n* = 374 763/63 766 671) in 2024–2025.

### AHC uptake

Total AHC uptake was compared across each completed QOF year, as summarised in Figure 2 and Supplementary Table S2. Among adolescents, uptake rose slightly from 68.08% (*n* = 15 940/23 414) to 71.58% (*n* = 19 217/26 846), whereas among adults uptake rose from 79.07% (*n* = 226 701/286 714) to 80.71% (*n* = 248 449/307 827). Despite these improvements, uptake among 14–17-year-olds continues to lag behind adults — 9.13% lower in 2024–2025. The vast majority of those who received AHCs were also recorded as having a health action plan in place, and the percentages of patients recorded as declining AHCs were relatively low (between 4.97% [*n* = 14 245/286 714] and 6.35% [*n* = 1593/25 082]). Monthly patterns showed a clear annual cycle, with the majority completed between January and March in the run up to the end of the QOF year (see Supplementary Figure S1).

**Figure 2.**
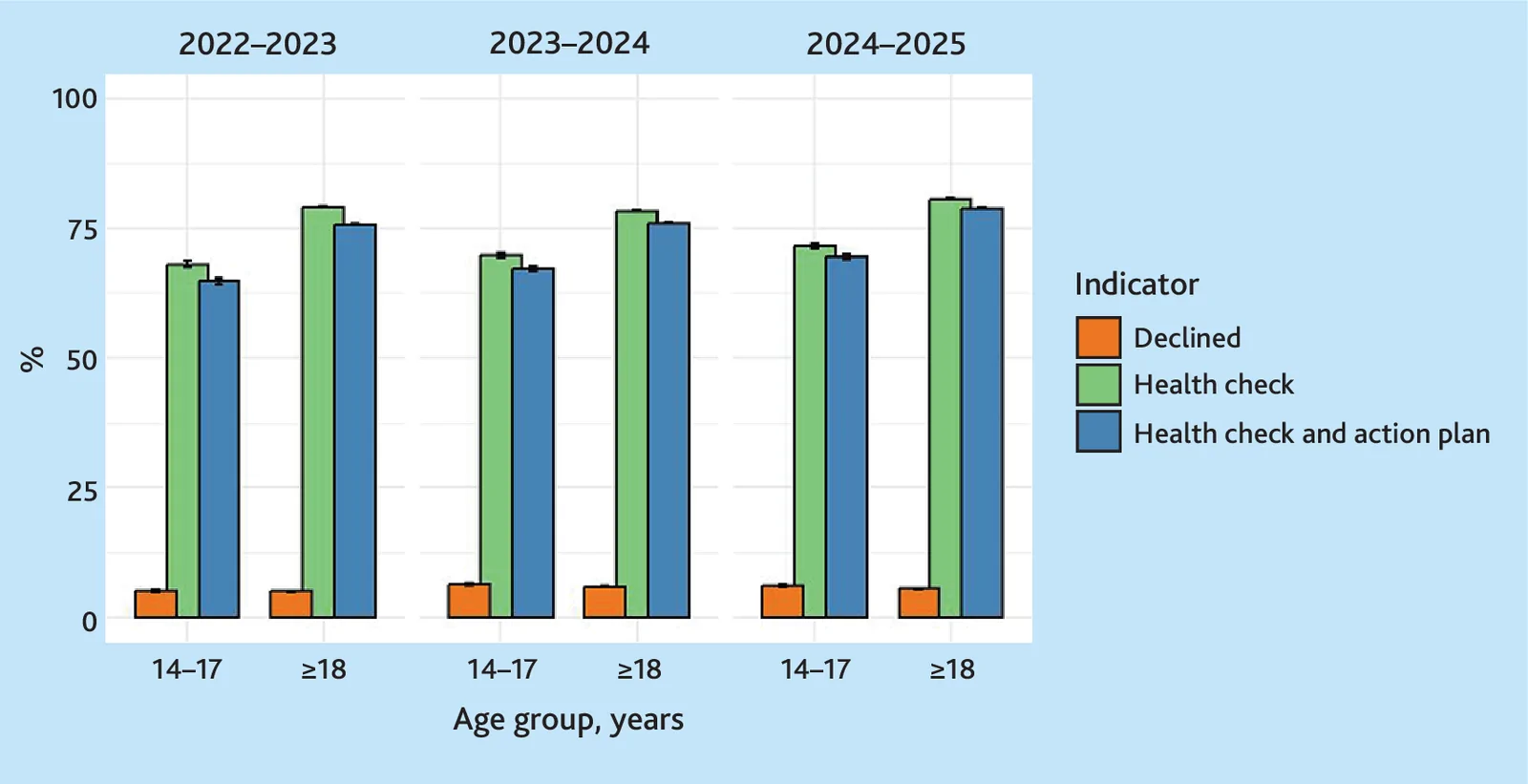
Percentages of 14–17-year-olds and adults on learning disability registers who received (with and without an action plan) an annual health check or declined an annual health check during each Quality and Outcomes Framework year. Error bars show Wilson 95% confidence intervals.

AHC uptake was only weakly correlated with the percentage of total QOF points the practice received that year among both 14–17-year-olds (*r*_s_ = 0.08) and adults (*r*_s_ = 0.17), indicating that uptake is not merely reflective of overall QOF performance.

### Regional differences in AHC uptake

Uptake of AHCs varied between regions of England (Figure 3 and Supplementary Table S3). Uptake was highest in London and lowest in the South West and East of England. However, these disparities narrowed in recent years, with uptake improving in all regions except for London, where uptake plateaued among adults and declined slightly among 14–17-year-olds.

**Figure 3.**
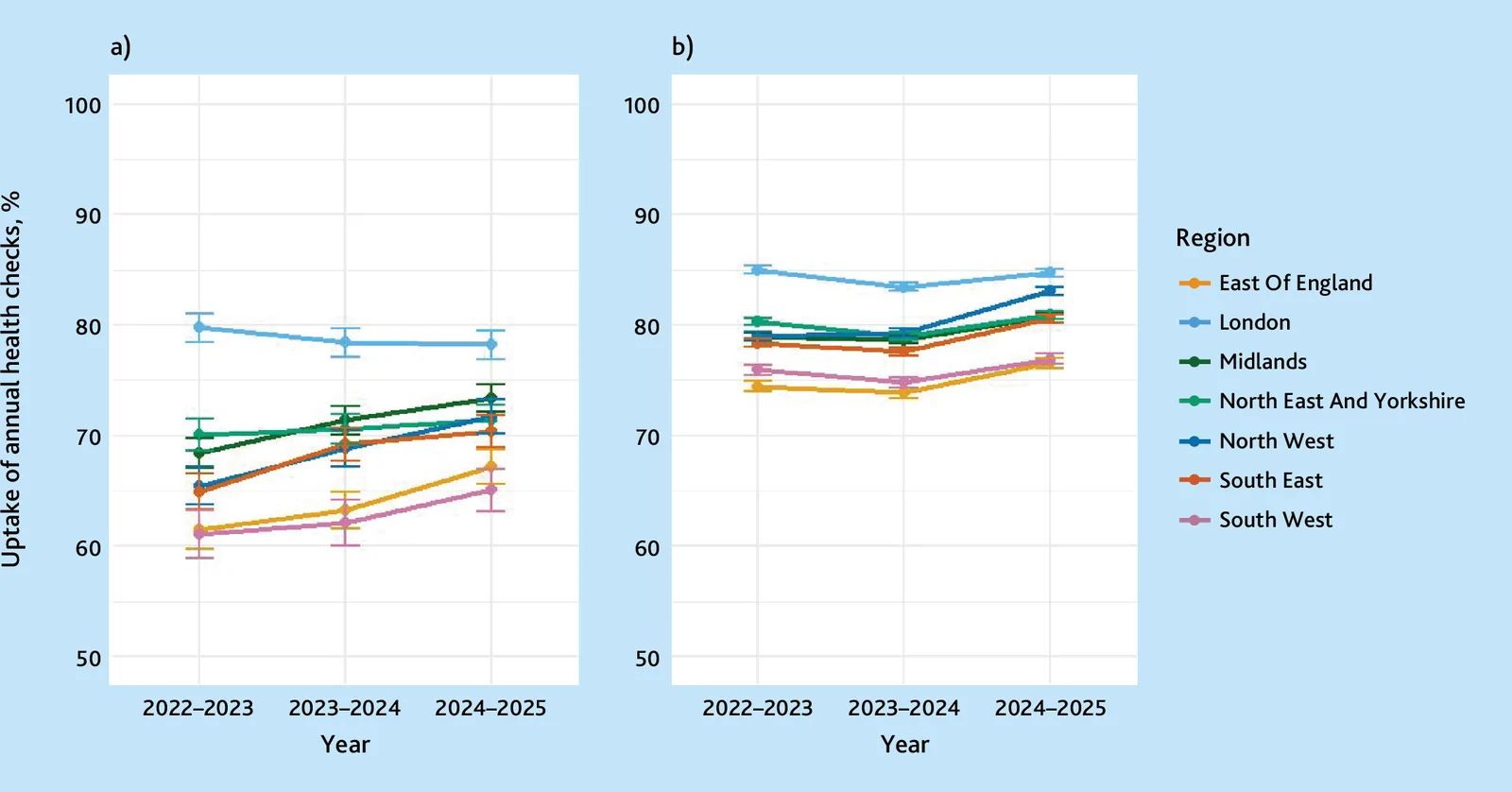
Percentage uptake of annual health checks among a) 14–17-year-olds and b) adults on the learning disability register between geographic regions in 2022–2025. Error bars show Wilson 95% confidence intervals.

### Neighbourhood deprivation and AHC uptake

There were small variations in AHC uptake across IMD deciles; for example, slightly higher uptake among adolescents in lower IMD deciles (Figure 4 and Supplementary Table S4). However, these differences were modest and do not indicate a strong relationship between neighbourhood deprivation and AHC uptake.

**Figure 4.**
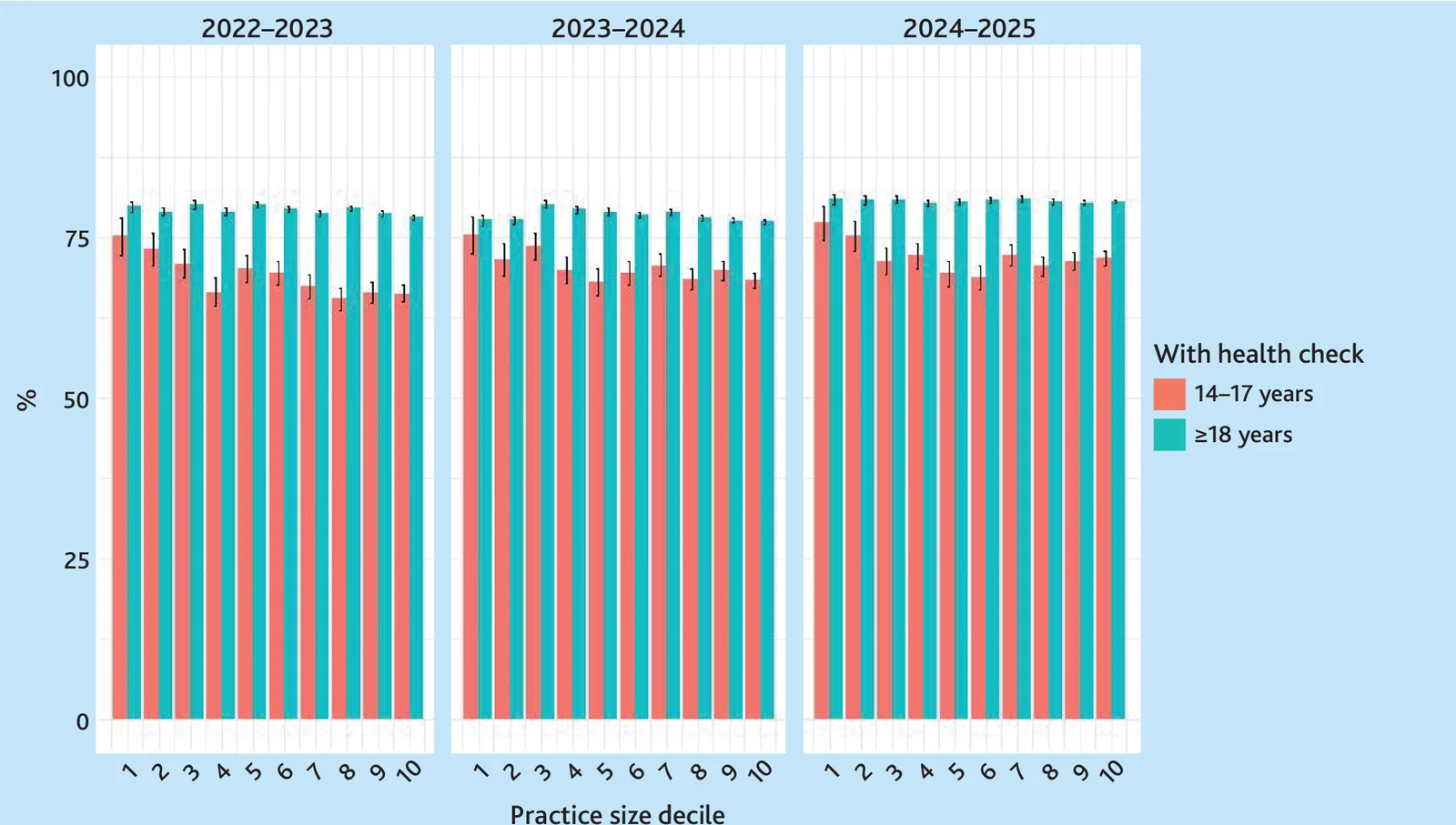
Learning disability health check uptake by practice Index of Multiple Deprivation decile from 2022 to 2025 (decile 1 = most deprived). Error bars show Wilson 95% confidence intervals.

### Practice size and AHC uptake

Figure 5 shows the uptake of AHCs by practice size decile. These indicate that uptake among 14–17-year-olds on the learning disability register was slightly higher in smaller practices whereas uptake among adults on the learning disability register was similar across all practice size deciles. The full data are summarised in Supplementary Table S5.

**Figure 5.**
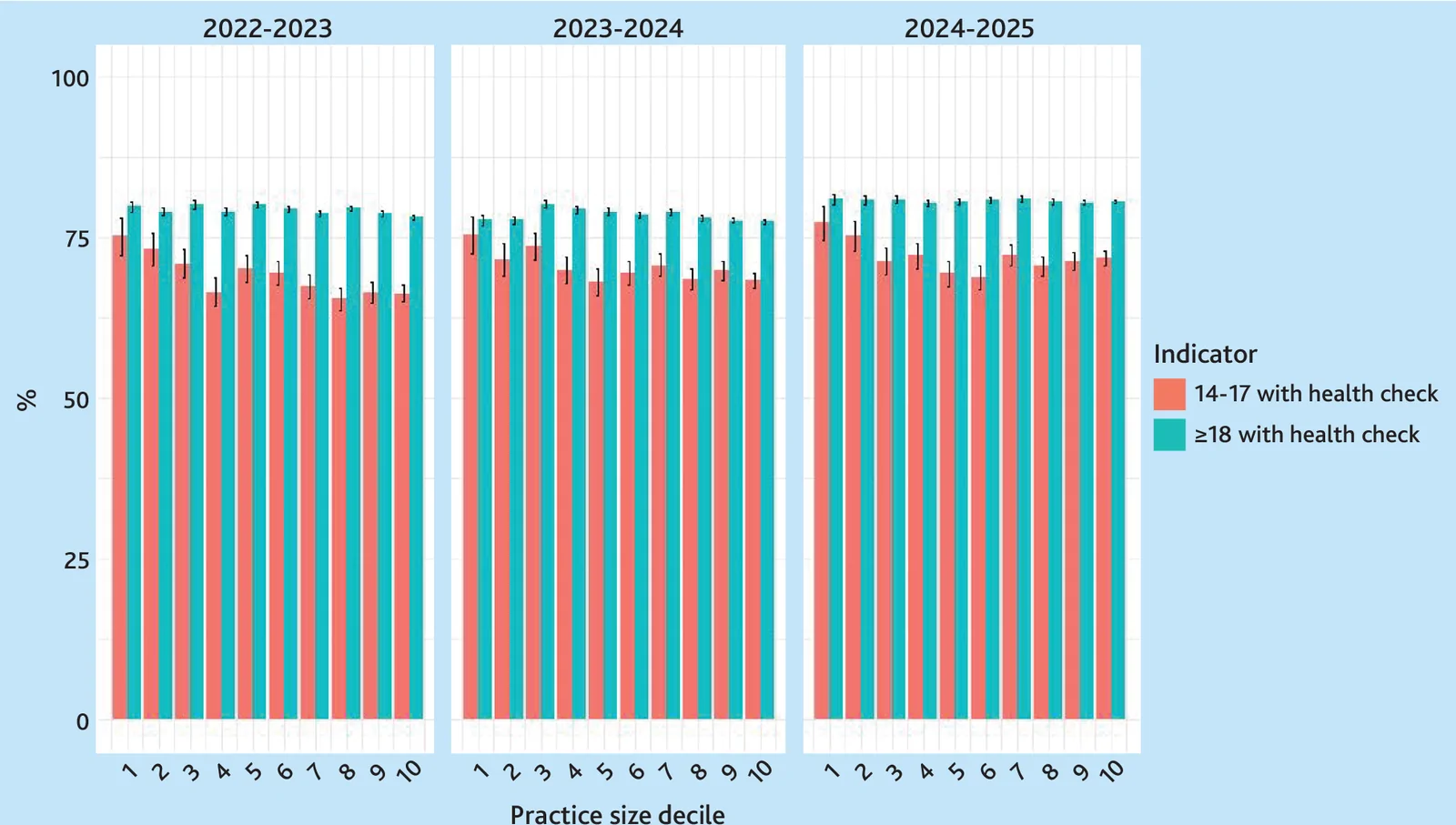
Learning disability health check uptake by practice size decile from 2022 to 2025. Error bars show Wilson 95% confidence intervals.

### Practice-level variability in AHC uptake

Figure 6 visualises practice-level variation in AHC uptake among 14–17-year-olds and adults during the 2024–2025 QOF year, alongside the number of eligible patients per practice. This highlights large variations in uptake among adolescents, with only 56.71% (*n* = 3012/5311) of practices reaching the NHS target of 75% (only including practices with ≥1 patient aged 14–17 years on learning disability register).^22^ The mean uptake among 14–17-year-olds was 71.43% and the median was 80.0%, but uptake was highly variable (standard deviation [SD] 31.39%, interquartile range [IQR] 50.0%). However, it should be noted that many practices had very few patients aged 14–17 years on learning disability registers, which contributes to the extreme distribution, with many practices with 0% or 100% uptake.

**Figure 6.**
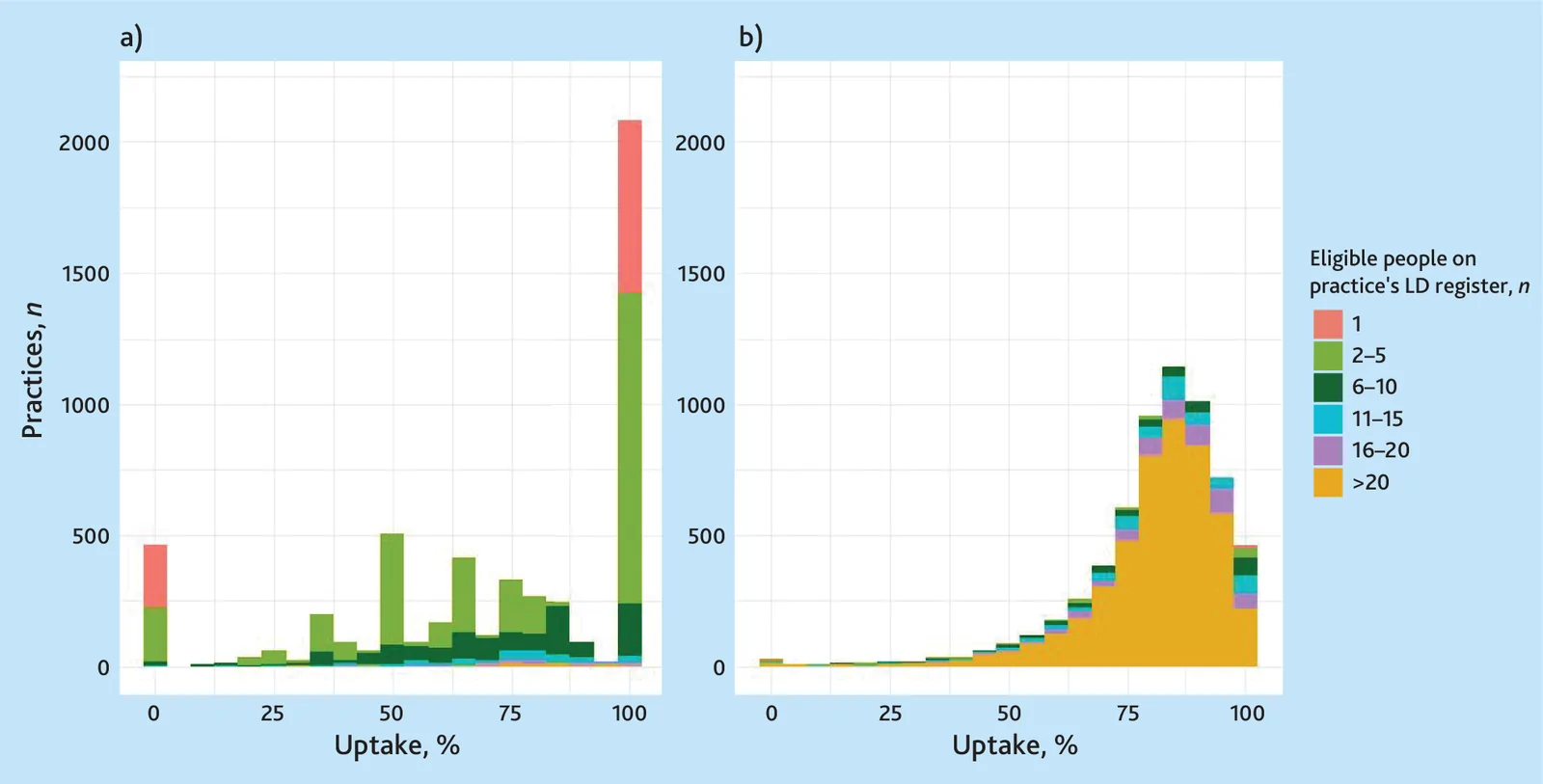
Histogram of the percentage uptake of annual health checks among a) 14–17-year-olds and b) adults between practices. LD = learning disability.

For adults, the mean uptake across practices was 80.68% and median was 83.78%. Uptake was also more consistent between practices among adults (SD 15.48%, IQR 15.63%). Although there are clearly still practices with low uptake, in 2024–2025, 75.81% of practices achieved the NHS target of 75%.

## Discussion

### Summary

Since 2022 the number of patients on GP learning disability registers has continued to increase and there have been modest improvements in AHC uptake, although uptake varies between age groups and geographic regions.

### Strengths and limitations

A strength of this analysis is its use of nationally representative data from almost all English general practices. This means that it provides comprehensive and up-to-date information to inform policy and research. All the data and code are also publicly available, allowing easy reproduction of the findings. However, the dataset includes only practice-level data relating to key indicators. This precludes examination of what factors might explain the temporal trends, regional disparities, and variability between practices. This also means that although it is known that learning disability registers miss many patients, it was not possible to examine characteristics of patients on learning disability registers that might help to understand which patients are being missed.

Another limitation is that there is considerable uncertainty in the precise number of patients registered at general practices and on learning disability registers owing to issues such as patients being registered at several practices. These data-quality issues mean that the precise percentage of patients on learning disability registers should be interpreted cautiously.

### Comparison with existing literature

These data suggest that efforts to improve the identification of people with a learning disability may be showing some progress. However, even with this increase, learning disability registers are still dramatically lower than national learning disability prevalence estimates of 1.5%–2%,^8,9^ indicating that many patients who may be eligible for AHCs are not being identified in primary care. This is likely because of multiple factors including under-ascertainment of people with milder learning disabilities, use of incorrect clinical codes in health records, and poor communication between specialist learning disability and primary care services.^9^

The modestly improved uptake of AHCs found in the current study continues a trend of gradual increases over the past decade.^13^ Encouragingly, the NHS target of 75% uptake now appears to be consistently met at a national level for adults. However, this threshold has never been reached for 14–17-year-olds, highlighting persistently lower uptake among younger patients.

The analysis in the current study also identified unequal implementation across England. In 2024–2025, adolescents in the South West were 13.11% less likely to receive an AHC than those in London, and adults in the East of England were 8.21% less likely to receive one than adults in London. However, in contrast, the current study found little evidence of meaningful differences in uptake across either practice size deciles or IMD deciles.

### Implications for research and practice

These data provide a helpful benchmark for evaluating changes in the identification of people with a learning disability in primary care and uptake of AHCs. Concerns have recently been raised about the possible de-prioritisation of learning disability AHCs. Despite the 2019 NHS Long Term Plan’s commitment to improving uptake of learning disability AHCs,^23^ similar commitments are notably absent from the recent Department of Health and Social Care’s *Fit for the Future: 10 Year Health Plan for England*.^24^ Moreover, changes have been implemented in the 2025–2026 QOF year whereby although learning disability AHCs remain incentivised and patients must still be on learning disability registers to be invited to an AHC, merely maintaining learning disability registers will not be directly incentivised.^25^ The impact of these changes is yet to be seen but it is important that learning disability AHCs remain a priority to help address learning disability health inequalities and prevent avoidable admissions.^12^

The current findings highlight those for whom increasing uptake is a particular priority — namely 14–17-year-olds and regions such as the South West and East of England. There are numerous factors that might contribute to the lower uptake among 14–17-year-olds, including lower awareness of AHCs among family carers, fewer health concerns, health needs being met by specialist paediatric services, or negative previous primary healthcare experiences. Similarly, there are many potential contributors to regional differences, including local policy and service landscapes. Unfortunately, a lack of research precludes richer understanding of the mechanisms underlying these differences and therefore potential solutions.

Although some progress appears to have been made with increasing the identification of people with a learning disability, primary care professionals should remain mindful that large numbers of patients with learning disabilities are likely to be missed. It is therefore important that general practices continue efforts to identify patients with learning disabilities and add them to learning disability registers to ensure they receive access to services such as AHCs. To aid in this, NHS England have developed guidance on improving the identification of people with a learning disability in primary care, including a ‘learning disability register inclusion tool’ with questions to help GPs consider whether it may be appropriate to add a patient to learning disability registers and codes that may indicate the presence of a learning disability.^26^

The national datasets used for this analysis are of great value for representatively characterising trends but are limited for understanding the causes underlying these. First, more qualitative research with people with a learning disability, family carers, and primary care professionals is needed to understand the experiences of AHCs and barriers to attendance.^27^ Second, more detailed quantitative research is needed to identify clinical and demographic predictors of uptake, understand factors contributing to regional disparities, examine the experiences of people with learning disabilities in the GP Patient Survey, and to continue to evaluate the impact of AHCs. In particular, although there is compelling evidence that AHCs lead to the identification of health problems and initiation of treatment,^12^ and some evidence for reduced hospital admissions,^11^ evidence is generally weaker regarding the longer-term health impacts of AHCs, and is especially lacking for young people. Finally, although nationally there are a range of local efforts to improve AHC uptake, these should undergo more rigorous evaluation and dissemination of their impacts to inform appraisal of their effectiveness and inform evidence-based policymaking.

## Data availability

The raw, monthly data releases from the Learning Disabilities Health Check Scheme can be downloaded from https://digital.nhs.uk/data-andinformation/publications/statistical/learning-disabilities-health-check-scheme/supportinginformation. An analysis-ready copy of the data in a single file, along with the accompanying R code, can be downloaded from https://osf.io/cf9kw/files/osfstorage.
